# Low Misrepresentation Rates of Scholarly Work in Otolaryngology-Head and Neck Surgery Residency Applications

**DOI:** 10.7759/cureus.6911

**Published:** 2020-02-07

**Authors:** Mohamedkazim Alwani, Morgan Sandelski, Lauren Van Buren, Elhaam Bandali, Jonathan Ting, Taha Shipchandler, Elisa A Illing

**Affiliations:** 1 Otolaryngology, Indiana University School of Medicine, Indianapolis, USA; 2 Otolaryngology, Indiana University School of Medicie, Indianapolis, USA; 3 Public Health, Richard M. Fairbanks School of Public Health, Indianapolis, USA

**Keywords:** misrepresentation, research, electronic residency applications

## Abstract

Objectives

To evaluate research trends, including rates of misrepresentation of scholarly work, in otolaryngology residency applications received by a single institution during the 2018-2019 residency application cycle.

Methods

After obtaining Institutional Review Board approval, all residency applications to the Department of Otolaryngology-Head and Neck Surgery at Indiana University School of Medicine, Indianapolis, IN for the 2018-2019 cycle were de-identified and analyzed. Demographic and research information including the number of listed peer-reviewed articles/abstracts, types of research projects, and misrepresentations were retrospectively evaluated.

Results

Our institution received 321 applications, which represented 69.5% of the entire 2018-2019 otolaryngology applicant pool. The average United States Medical Licensing Examination (USMLE) Step 1 score was 246 ±12.4. There were 203 (62.2%) applicants who reported 591 published citations with 20 (6.2%) applicants misrepresenting 26 items (4.4%). Applicants who misrepresented research output had lower average Step 1 scores (237.4 vs 246.4, p: <0.05). Self-promotion to higher authorship status was the most common form of misrepresentation (61.5%).

Conclusions

The role of scholarly work in stratifying applicants continues to expand. Although a competitive application climate motivates a minority of applicants to misrepresent scholarly work, rates of misrepresentation in otolaryngology applications are low and continue to decline. The level of evidence assigned to this study is III.

## Introduction

Otolaryngology remains a competitive program in the National Residency Matching Program (NRMP). In the 2018-2019 application cycle, 462 applicants competed for 328 spots [1]. Average United States Medical Licensing Examination (USMLE) scores have continued to rise, with the average USMLE Step 1 score of US seniors matched into otolaryngology rising from 243 in 2011 to 248 in 2018 [2,3]. This demonstrates a competitive match climate attributed to the availability of limited training spots despite growing interest from capable applicants [4].

Since high test scores and clerkship grades have become the expectation, the pursuit of additional academic endeavors has become more important in improving competitiveness [5]. At the same time, an increase in the number of publications has become evident among applicants, indicating either an increase in academic productivity due to competition or an increased rate of embellishment [6]. Unlike standard metrics employed in tests, there is no standardized approach to evaluate/verify applicant research work. Despite the introduction of a categorized reporting system implemented under the Electronic Residency Application System (ERAS), the lack of a validation tool provides applicants with latitude to misrepresent scholarly accolades given that applicants may feel compelled to overstate their accomplishments to stand out as more proficient candidates [4,7]. Although misrepresentation of research work has been demonstrated across multiple specialties, it has not been investigated in otolaryngology residency applications recently [4,6-13].

Given the expanding role of scholarly activity in stratifying applicants and the paucity of information regarding this subject, our study aimed to evaluate trends and misrepresentations in the reported research activity of otolaryngology residency applicants to our institution.

## Materials and methods

After gaining approval from our Institutional Review Board, ERAS applications submitted to the Otolaryngology-Head and Neck Surgery residency program at our institution for the 2018-2019 cycle were reviewed. Applicant demographics and research activity were extracted. Research activity was categorized into two groups: “published peer-reviewed journal articles/abstracts” and “all other articles/abstracts.” Data endpoints related to applicant research activity were extracted only from the former group. All items categorized as “published peer-reviewed journal articles/abstracts” were verified using PubMed, Google Scholar, Ulrich’s Periodicals Directory, individual journal websites, and individual online sources for correct reporting. Published articles/abstracts were categorized by type. Misrepresentation was defined in accordance with criteria established by Meeks et al. with minor modifications as follows: (1) non-authorship of a published article in which authorship was claimed, (2) claimed authorship of non-existent/unverifiable article, (3) self-promotion to a higher authorship status within a published article, and (4) non-peer reviewed article/abstract reported as peer-reviewed [7]. The number of podium and poster presentations were recorded for applicants but were not included in misrepresentation analyses.

Demographic and select applicant research data were summarized using descriptive statistics. Categorical variables were evaluated using Fisher’s exact test or Pearson’s chi-squared test when appropriate, using GraphPad Prism v8.0 (GraphPad Software, San Diego, CA). Statistical significance was defined as a p-value of <0.05.

## Results

Demographics 

Our institution received 321 applications, which represented 69.5% of the entire 2018-2019 otolaryngology applicant pool. The average age of applicants was 27 years, with the youngest applicant being 23 years in age and the oldest 45 years. There were 124 (38.6%) applicants who identified as female. Most applicants were American medical graduates (AMGs) while 19 (5.9%) applicants were international medical graduates (IMGs). There were 19 (5.9%) applicants from Doctor of Osteopathy (DO) programs. Of the 258 (80.4%) who were eligible for Alpha Omega Alpha (AOA), 101 (39.2%) applicants were AOA members. There were 63 (19.6%) who were not eligible for AOA, with 13 of these applicants having elections after applications were due and 50 coming from schools without AOA programs. There were 243 (75.7%) applicants who went to a medical school that used honors or an equivalent mark of high mastery, and 152 (62.6%) of those applicants had achieved honors on the surgery core clerkship (Table 1).

**Table 1:** Applicant demographic details

Characteristics	
Male, n (%)	197 (61.4)
Female, n (%)	124 (38.6)
Mean age, years	27
American medical graduates, n (%)	302 (94.1)
International medical graduates, n (%)	19 (5.9)
Doctor of Medicine (MD), n (%)	302 (94.1)
Doctor of Osteopathy (DO), n (%)	19 (5.9)
Alpha Omega Alpha status, n (%)	101 (39.2)
Honors in surgery clerkship, n (%)	152 (62.6)

The average USMLE Step 1 score was 246 ±12.4. There were 223 (69.5%) applicants reporting a USMLE Step 2 Clinical Knowledge (CK) score, with an average of 252 ±12.2. The average Step 1 score of students who reported a Step 2 CK score was 243 compared to an average Step 1 score of 253 for applicants who did not report Step 2 CK score (p: <0.0001).

Research trends

**Figure 1:**
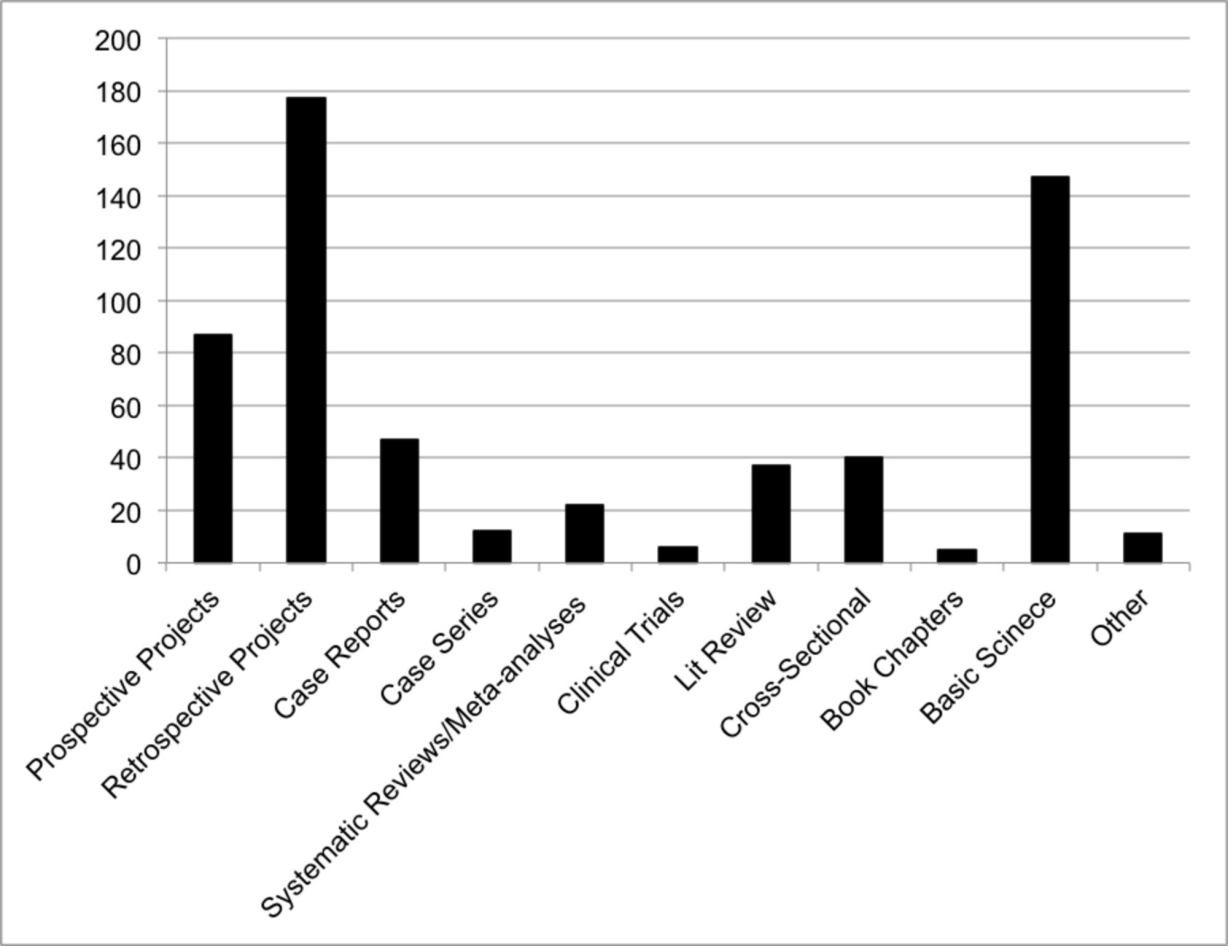
Frequency distribution of different types of published peer-reviewed journal articles/abstracts

Of the 1,236 items listed by 272 (84.7%) applicants, only 591 items were listed as published peer-reviewed journal articles/abstracts, by 203 (62.2%) applicants. The most common article types were retrospective studies (177, 29.9%) followed by basic science studies (147, 24.9%). Articles reported under the “other” category included three proofs of concept, one opinion article, one viewpoint article, one experience article, and five uncategorized articles. A total of 680 oral presentations and 1,195 poster presentations were reported by 224 (69.8%) and 285 (88.8%) applicants, respectively. There was an average of 2.1 oral presentations and 3.7 poster presentations per applicant (Figure 1).

There were 64 (19.9%) applicants with a history of dedicated research time, with a mean duration of 1.8 years. A total of 21 (31.3%) applicants procured dedicated research time prior to medical school, 31 (48.4%) during medical school, 10 (15.6%) after medical school, and 3 (4.7%) took time during and after medical school. There was a significantly higher mean number of published peer-reviewed journal articles/abstracts (3.4 vs 1.5, p: <0.0001), oral presentations (3.2 vs 1.8, p: <0.001), and poster presentation (4.8 vs 3.4, p: <0.01) for applicants who procured dedicated research time (Table 2). 

**Table 2:** Types of publication by dedicated research time vs. none *Statistically significant since p-value was <0.05

	Dedicated research time, n (%)	None, n (%)	P-value
Prospective projects	30 (13.9%)	57 (15.1%)	0.7192
Retrospective projects	64 (29.6%)	113 (29.9%)	>0.9999
Case reports	13 (6.0%)	34 (9.0%)	0.2105
Case series	4 (1.9%)	8 (2.1%)	0.9999
Systematic reviews and/or meta-analyses	10 (4.6%)	12 (3.2%)	0.3741
Clinical trials	3 (1.4%)	3 (0.8%)	0.6732
Literature review	13 (6.0%)	24 (6.4%)	>0.9999
Cross-sectional	7 (3.2%)	33 (8.7%)	0.0102*
Book chapters	2 (0.9%)	3 (0.8%)	>0.9999
Basic science	65 (30.1%)	82 (21.7%)	0.0294*
Other	4 (1.9%)	7 (1.9%)	>0.9999
Total articles published	215	376	

When stratifying applicants by Step 1 scores (<240, 240-259, and >260), there were no significant differences in publications (F = 0.07, p: >0.05), poster presentations, (F = 1.0, p: >0.05), or oral presentations (F = 2.8, p: >0.05). 

Misrepresentation of research work

**Figure 2:**
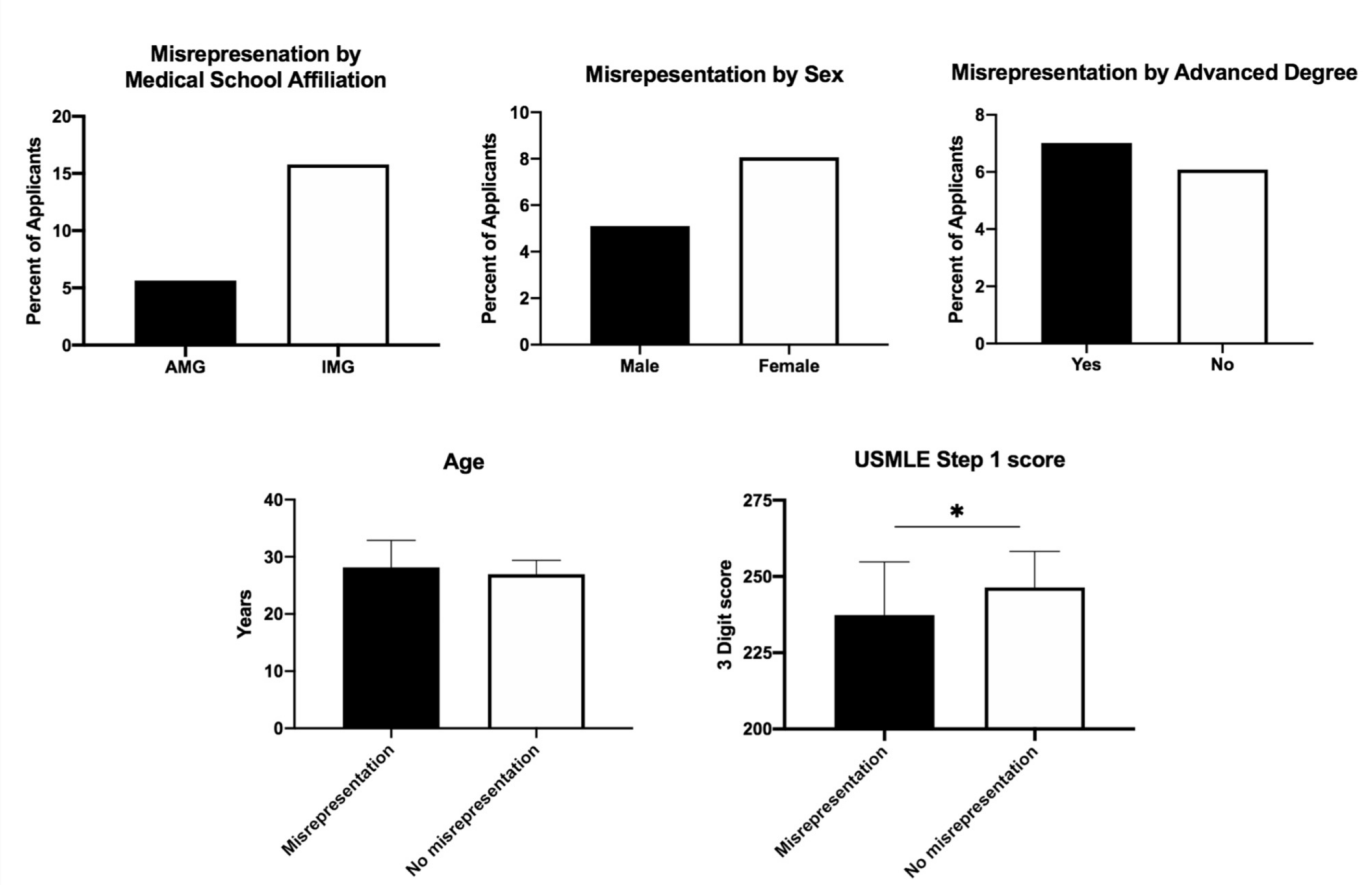
Distribution of misrepresentation by category *Statistically significant since p-value was <0.05

There were 26 (4.4%) articles misrepresented by 20 (6.2%) applicants. The most common type of misrepresentation was self-promotion to a higher authorship status (61.5%), followed by non-peer reviewed items listed as peer-reviewed articles/abstracts (30.8%). The average age of applicants who misrepresented their research was not significantly different from those who did not (28.1 vs 27.0, p: >0.05). Of the 20 applicants, three (15.8%) were IMGs and 17 (5.7%) were AMGs (p: >0.05). Applicants' sex did not predict an increase in the rates of misrepresentation [10 males (5.1%) versus 10 females (8.1%), p: >0.05)]. Similarly, having an advanced degree also did not predict misrepresentation (7.0% vs 6.1%, p: >0.05). However, applicants who misrepresented research output had a lower average Step 1 score than those who did not (237.4 vs 246.4, p: <0.05) (Figure 2)

## Discussion

Similar to other surgical subspecialties, otolaryngology has traditionally seen highly competitive residency match rates as evidenced by a higher number of proficient applicants compared to the number of training positions available [1]. This application climate has required medical students to demonstrate progressively higher levels of academic accomplishment. Successful applicants have demonstrated higher scores on standardized tests as well as increased scholarly productivity. A comparison between 2011 NRMP and 2018 NRMP statistics has revealed that the mean number of abstracts, presentations, and publications from accepted otolaryngology applicants has doubled from 5.1 to 10.4 [2,3].

An increasingly qualified applicant pool poses a difficult challenge for faculty involved in screening/selecting applicants [7]. As a result, scholarly activity is now routinely used as an important stratification metric since this is a proxy of an applicant’s diligence at task completion, work ethic, and academic potential during residency [6,7]. Unfortunately, the highly competitive nature of applying to surgical subspecialties may motivate applicants to artificially enhance their academic records so that they would be viewed more favorably [6,8].

Reports regarding misrepresentation of scholarly work in residency applications have shown significant variability with rates ranging from 1% to 45% [8]. Reasons for such variability include varying definitions of misrepresentation, evaluation of different applicant pools, and application of different search criteria and verification protocols [6,14]. Despite this variability, rates of misrepresentation of scholarly work in surgical subspecialty applications have typically been lower than average. A recent study by Meeks et al. found 13 cases of misrepresentation (1.18%) among 1,100 citations listed by 323 published applicants from a total pool of 573 applicants to a single orthopedic surgery program [7]. Similarly, Cohen-Gadol et al. found 9 cases of misrepresentation (6%) among 212 citations listed by 73 published candidates from a total pool of 102 applications to a single neurosurgical residency program [14].

The rate of misrepresentation in otolaryngology residency applications has not been investigated recently. In 2010, Beswick et al. reported 22 cases of misrepresentation (5.1%) among 432 citations listed by 173 published applicants from a total pool of 325 applications [4]. Our study found 26 cases of misrepresentation (4.3%) among 591 citations listed by 203 published applicants from a total pool of 321. Our results demonstrate a decrease in misrepresentation rates similar to results published by Meeks et al. for a single orthopedic residency program [7]. This decline may be attributed to features introduced on ERAS by the Association of American Medical Colleges (AAMC). One such feature is the provision of nine different categories for publication types, which provides a structured framework for applicants to list their scholarly work and removes the ambiguity that results from freehand self-reporting [6]. Additionally, in the era of web searchability, the requirement of PubMed Identifiers (PMID) for peer-reviewed journal articles/abstracts has decreased the latitude for applicants to misrepresent academic work [7]. Alternatively, this decline in misrepresentation rates can also be attributed to increasing academic integrity among otolaryngology applicants due to appropriate mentorship and positive examples.

Similar to results published by Beswick et al., a noteworthy finding from our study was the higher rates of misrepresentation found in applications of medical students with lower test scores (237.4 vs 246.4, p: <0.05) [4]. However, in contrast to results published by Meeks et al., we did not identify increased rates of misrepresentation in the IMG sub-group [7]. This finding, in turn, is consistent with studies of applicants to an emergency medicine residency program and a gastroenterology fellowship program, both of which showed no difference in misrepresentation rates between IMGs and AMGs [9,15]. Additionally, age, gender, and advanced degrees did not differ significantly between the misrepresenting and non-misrepresenting group.

In contrast to the findings of Beswick et al. on misrepresentations in otolaryngology residency applications, which showed that unverifiable publications constituted the most common type of misrepresentation, our cohort demonstrated only two citations that were unverifiable [4]. Lower numbers of unverifiable citations are likely due to advances in journal/article indexing and improved web search functionalities. Instead, our cohort demonstrated that author promotion was the most common type of misrepresentation. This is similar to findings reported by Meeks et al. and possibly represents a subtler way of exaggerating academic work [7].

One of the main limitations of our study was the inclusion of only published peer-reviewed journal articles/abstracts. Since we did not evaluate “submitted”, “provisionally accepted”, “accepted”, and “in-press citations”, our misrepresentation rates may have been underestimated given that these categories collectively represent items that are most at risk of being misrepresented. However, since ERAS does not provide any definitions for the above-mentioned items, misrepresentations may represent a misinterpretation of these terms by research-naïve medical students rather than intentional inflation of academic work. Additionally, given that “submitted” articles were not prospectively evaluated, the fate of these articles was not determined. An interesting finding reported by Pak et al. demonstrates that of all “submitted” articles, 44% (110 out of 250) remained unpublished after 12 months. Additionally, of the 140 articles that did get to publication, 40.7% of articles were published in a lower-impact journal compared to the original journal of submission [8].

In addition to the aforementioned findings, the progressively declining rates of misrepresentation is a testament to the academic integrity of otolaryngology applicants, and an indication of a bright future for our specialty. This improvement also represents the outcome of appropriately designed updates to ERAS. However, the authors recommend that ERAS provide applicants with definitions for the terms “submitted”, “provisionally accepted”, “accepted”, and “in-press” to minimize the risk of misinterpretation.

## Conclusions

The role of scholarly work in stratifying applicants has continued to gain prominence. Although the highly competitive milieu of the application may motivate a small minority of applicants to misrepresent scholarly work, the rates of misrepresentation in otolaryngology are low to begin with and are on a progressive declining trend.
